# Editorial: The expanding Frontiers of stem cells therapy in oral maxillo-facial engineering and regenerative medicine

**DOI:** 10.3389/fbioe.2025.1548950

**Published:** 2025-01-22

**Authors:** Christiane L. Salgado, Andrea Cochis, Elena M. Varoni, Rui Amaral Mendes

**Affiliations:** ^1^ i3S-Instituto de Investigação e Inovação em Saúde da Universidade do Porto, Porto, Portugal; ^2^ INEB-Instituto Nacional de Engenharia Biomédica, Universidade do Porto, Porto, Portugal; ^3^ Department of Health Sciences, Center for Translational Research on Autoimmune and Allergic Diseases CAAD, Università del Piemonte Orientale UPO, Vercelli, Italy; ^4^ Department of Biomedical, Surgical and Dental Sciences, Milan State University, Milan, Italy; ^5^ Department of Community Medicine, Information and Health Decision Sciences (MEDCIDS), Faculdade de Medicina da Universidade do Porto, Porto, Portugal; ^6^ RISE-Health, PerMed Research Group, Faculdade de Medicina da Universidade do Porto, Porto, Portugal; ^7^ RISE-Laboratorio Associado, LT2-Clinical and Translational Research in Oncology, Faculdade de Medicina da Universidade do Porto, Porto, Portugal; ^8^ Department of Oral and Maxillofacial Medicine and Diagnostic Sciences, Case Western Reserve University, Cleveland, OH, United States

**Keywords:** oral and maxillo-facial applications, dental MSC and extracellular vesicles applications, dental stem cell-based therapy, dental MSC delivery technology, dental/mucosa/bone tissue engineering, scaffold for dental/mucosa/bone tissue engineering, oral biology, antibacterial properties

The reconstruction of oral and maxillofacial tissues is a complex challenge with significant socioeconomic and health impact, as these structures are critical for both function and aesthetics (Shetty et al., 2024). Current clinical strategies often fail due to limitations in mechanical stability, regenerative potential, and immunomodulation (key biological factors), while the risk of infection remains a major obstacle to reach a successful tissue healing (Li et al., 2024). These challenges underscore the urgent need for innovative, multidisciplinary approaches in tissue engineering and regenerative medicine.

Stem cell-based therapies, particularly those utilizing dental-derived mesenchymal stem cells (dMSCs) and their bioproducts, have emerged as promising tools for advancing oral and maxillofacial tissue regeneration (Li et al., 2021). dMSCs, including those derived from dental follicle sac, offer distinct advantages due to their easy access, high proliferative capacity, and strong differentiation potential into osteogenic, chondrogenic, and neurogenic lineages (Salgado et al., 2020). Additionally, their immunomodulatory properties help regulate inflammation, creating a conducive environment for tissue repair (Costa et al., 2023). In the work of Souto-Lopes et al., authors evaluated the *in vitro* osteoinduction of periodontal-originating cells (human dental follicle mesenchymal cells - DFMSCs) promoted by a nano-hydroxyapatite/chitosan (nHAp/CS) bioaerogel. They also evaluated *in vivo* the bone regenerative capacity of the biocomposite assessed by a rat calvaria critical bone defect model. The results demonstrated the bioaerogel had an *in vitro* favorable condition for DFMSC proliferation and osteogenic differentiation, while *in vivo* showed higher bone ingrowth. This low environmental impact composite produced by an eco-friendly process could be further explored as an alternative biomaterial for guiding alveolar bone tissue regeneration.

Therefore, the improvement of innovative bioactive biomaterials can represent another promising strategy to boost the tissue healing by directly influence dMSC fate, leading to faster regeneration by the release of targeted cell derivates such as extracellular vesicles (EVs). The EVs from human MSC were studied by Huang et al., as a potential tool for therapeutic translation application, and authors had characterized their bioactivity before and after lyophilization as a potential tool to preserve them for further clinical application due to their instability as well as their suitability for bone regeneration in stimulating the BMP-2 cascade *in vitro* and *in vivo*. Results highlighted the ability of DMSO acting as a powerful cryopreserving agent prior to lyophilization to maintain the functional stability of the engineered EVs and serve as a baseline for its potential in clinical therapies. Authors intend for future work a long-term stability evaluation of these DMSO-lyophilized EVs and their potential applicability to different tissues and delivery mechanisms.

Another smart strategy to produce bioactive scaffolds involves a multifaceted approach to personalize patients’ treatments during the materials synthesis that can provide sustained and controlled agent release, increasing the efficacy against pathogens, preventing inflammation and infection *in situ*. The study of Moreno-Flores et al. aimed to evaluate the *in vitro* osteogenic behavior of human bone marrow stem cells and assess the antimicrobial response of 3D printed porous scaffolds produced with propolis-modified wollastonite. The porous scaffolds created through 3D printing with triply periodic minimal surface (TPMS) gyroid geometry exhibited mechanical properties suitable for repairing trabecular bone tissue. Additionally, impregnating the scaffolds with ethanolic extracts of propolis (EEPs) showed effective control over bacterial growth, and, in the same time, high bioactivity properties by the increase of bone marrow MSC’s proliferation by the reduction of the oxidative stress, thus reporting anti-inflammatory properties, too. This use of a natural product and its sustainable release opened new opportunities for further research, since the biomaterial kept the antimicrobial activity while minimized its impact on the cellular microenvironment as well as by reducing oxidative stress.

To study immunomodulation and anti-inflammatory response, the work of Zhang et al. investigated the possibility to regulate by siRNA the expression of phosphatase and tensin homolog (PTEN) that is critical for inflammatory regulation and osteogenic capacity to modify cell response, such as, adipose stem cell from type II diabetic rats (TADSCs), which exhibited an impaired osteogenic capacity. TADSCs showed upregulation of the PTEN mRNA expression, but when it was silenced by the specific PTEN siRNA, the expression of genes related to the AKT/mTOR/HIF-1α signaling pathway was renews thus promoting the macrophages’ M2 polarization and decreasing M1 polarization *in vitro*, as well. In the *in vivo* model, PTEN inhibition in TADSC sheets surrounding titanium implants promoted macrophage polarization toward the M2 phenotype, attenuating the inflammatory response, and enhancing the osseointegration, suggesting a potential therapeutic approach of modifying stem cells’ genetic signature derived from patients towards the PTEN downregulation to enhance bone regeneration.

Novel translation studies of craniofacial soft tissue injuries focus in the tissue reconstruction and repair using *in vitro* biofabrication of mature tissue constructs that can supply an alternative to the current clinical surgical standard. The work of Feinberg and Marcelo used small punch biopsies from the oral mucosa as source of the cells to promote lip reconstruction and function and improve patient´s rehabilitation and quality of life. The most important goal of lip regeneration is maintaining oral circumference with a viable, well-perfused microvascular flap with sufficient bulk to perform the necessary surgical repairs. This requires restoring continuity of the labial vestibule and orbicularis oris muscle (restoration of lips’ striated muscle volume), with an intact motor innervation. The best results occurred with a completely intact sphincter regeneration with an active motor function and sensory sensation. Authors have successfully developed an animal model and showed that it was possible to develop a prelaminated, innervated, pre-vascularized, prefabricated microvascular free flap for dynamic, functional reconstruction of complex, composite soft tissue defects of the lip.

Finally, precision and stratified medicine requires a pattern analysis of datasets that are key elements to investigate efficacy in medical treatments. Advanced statistical methods and machine learning techniques could identify patterns, revealing new hypotheses about disease mechanisms or treatment efficacy, as well as to match treatments and clinical revisions in order to develop predicative models supporting the clinicians’ decisions. By examining adverse events or treatment failures in a clinical population, researchers can identify gaps in existing therapies, leading to the optimization of treatment protocols or the development of novel therapeutic approaches and disease prevention so-called bed-to-bench side data.

The work of Barbaro et al. found correlation between the number of occlusal caries and anthropometric indexes predicting insulin resistance (IR) and showed that nutritional supplement myo-inositol (MYO) might antagonize the detrimental effects of IR on tooth decay. The work provided feasibility for clinical studies on MYO as a regenerative factor in dentistry and oral surgery, including dysmetabolic/aging conditions, bone reconstruction in oral destructive/necrotic disorders, dental implants, and for empowering the efficacy of a number of tissue engineering methodologies in dentistry and oral surgery. The used reverse translational approach enables the development of personalized treatment plans based on genetic, epigenetic, and phenotypic data derived from patient populations. A number of tissue engineering techniques for dental hard tissues and oral bone might find in MYO a new molecule for the amelioration of their reparative/regenerative action. In particular, they expected that MYO would electively target vascular and stromal/MSC-like cells of the dental/alveolar/oral bone tissues. By combining *in vivo*, *in vitro*, and *in silico* studies, authors concluded that MYO has the potential to be an effective, low cost, easy to deliver, and highly tolerable regenerative factor, thus, it could be used in tissue engineering of hard/mineralized tissues, contributing to personalized treatments in dentistry and oral surgery.

The advancements in stem cell therapy for regenerative solutions, particularly with MSCs and their bioproducts, are transforming the landscape of oral and maxillofacial tissue engineering. The manuscripts in this Research Topic collectively illustrate the transformative potential of stem cell-based therapies and biomaterials in addressing the challenges of oral and maxillofacial tissue engineering. By leveraging the unique properties of dental-derived mesenchymal stem cells (dMSCs), extracellular vesicles (EVs), and innovative scaffold designs, these studies highlight the convergence of advanced biomaterial science and regenerative medicine. In fact, dental-derived MSCs demonstrated remarkable potential for overcoming existing clinical challenges, including promoting tissue regeneration, enhancing mechanical stability, and modulating immune responses as well as to provide new insights suitable for *in silico* predictive models. Their versatility and ease of accessibility position them as pivotal tools in developing innovative therapeutic approaches. Likewise, the application of bioactive scaffolds and engineered EVs for targeted regeneration exemplifies how multidisciplinary approaches are tailoring solutions to overcome the clinical hurdles of mechanical stability, immunomodulation, and infection control.

Overall, these contributions not only advance the understanding of stem cell behavior and biomaterial interactions, but also offer scalable solutions for real-world challenges such as cost-effectiveness, sustainability, and accessibility.

Despite these promising developments, the path to clinical translation remains fraught with challenges that demand continued innovation and interdisciplinary collaboration, rigorous standardization, and comprehensive evaluation of treatment’s safety and efficacy. In fact, this Research Topic underscores the importance of integrating novel methodologies, such as reverse translational approaches and machine learning-based predictive models, to bridge the gap between bench research and bedside application. The integration of personalized treatment paradigms, sustainable biomaterial development, and computational tools has the potential to redefine the therapeutic strategies.

Moving forward, the collective insights presented in this Research Topic pave a clear trajectory for addressing the critical unmet needs in oral and maxillofacial regenerative medicine, setting the stage for more comprehensive, patient-centered, and efficient therapeutic solutions.
